# Genetic characterization of *Toxoplasma gondii* in Yunnan black goats (*Capra hircus*) in southwest China by PCR-RFLP

**DOI:** 10.1186/s13071-015-0673-0

**Published:** 2015-01-27

**Authors:** Qiang Miao, Si-Yang Huang, Si-Yuan Qin, Xin Yu, Yan Yang, Jian-Fa Yang, Xing-Quan Zhu, Feng-Cai Zou

**Affiliations:** College of Animal Science and Technology, Yunnan Agricultural University, Kunming, Yunnan Province 650201 PR China; State Key Laboratory of Veterinary Etiological Biology, Key Laboratory of Veterinary Parasitology of Gansu Province, Lanzhou Veterinary Research Institute, Chinese Academy of Agricultural Sciences, Lanzhou, Gansu Province 730046 PR China; College of Animal Science and Technology, Jilin Agricultural University, Changchun, Jilin Province 130118 PR China

**Keywords:** *Toxoplasma gondii*, Genotype, PCR-RFLP, Yunnan black goats, China

## Abstract

**Background:**

*Toxoplasma gondii* is a protozoan parasite that infects almost all warm-blooded animals and human beings. Goats are one of the susceptible animals to *T. gondii*. However, little is known of genetic diversity of *T. gondii* in Yunnan black goats in China. The objective of this present study was to determine the genotypes of *T. gondii* isolates from black goats in Yunnan province, southwest China.

**Methods:**

Genomic DNA was extracted from liver (n = 403), lung (n = 403) and lymph nodes (n = 250) of Yunnan black goats and assayed for *T. gondii* infection by semi-nested PCR of B1 gene. Then, the positive DNA samples were typed at 10 genetic markers using polymerase chain reaction-restriction fragment length polymorphism (PCR-RFLP) technology. These markers include 9 nuclear loci, namely, SAG1, SAG2 (5’-SAG2 and 3’-SAG2, alternative SAG2), SAG3, BTUB, GRA6, c22-8, c29-2, L358, PK1, and an apicoplast locus Apico.

**Results:**

Out of 403 tested samples, 20 (4.96%) DNA samples were *T. gondii* positive by amplification of B1 gene. Among them, 2 isolates were genotyped at all loci, and 6 isolates were genotyped for 8 or more loci. In total, seven samples belong to ToxoDB PCR-RFLP genotype#10 (Type I), and one belongs to genotype ToxoDB #9.

**Conclusions:**

To our knowledge, this is the first report of ToxoDB#9 and ToxoDB#10 *T. gondii* in Yunnan black goats in China. These results revealed a wide distribution of these *T. gondii* in Yunnan black goats in China, which has important implications for public health.

## Background

*Toxoplasma gondii* is an obligate intracellular parasite, causing toxoplasmosis in almost all warm-blooded animals and humans [1]. Generally, *T. gondii* infection rarely causes clinical symptoms in healthy individuals, however, it can cause severe diseases, even fatal to AIDS patients or those individuals with cancer undergoing immuno-suppressive therapy [2].

Yunnan is a province having 25 different ethnic groups, where halal food like mutton is well-received for human consumption. Goats are commonly infected with *T. gondii* [1], and it can be a potential source for human toxoplasmosis through consumption of uncooked or raw mutton containing *T. gondii* tissue cysts [3]. In view of previous serology reports in Yunnan Province, seroprevalence of *T. gondii* infection was 21.6% [4], 17.0% [5], 27.1% [6], 12.6% [7], 19.9% [8], 6.3% [9] in pet dogs, pigs, equids, peafowls, black-headed gulls and goats, respectively, which revealed a widely distribution of *T. gondii* infection in this province. In addition, variable genotypes of *T. gondii* were identified from HIV positive patients [10], pigs [11], cats [12] and bats [13] in Yunnan Province. However, little information is available about the genetic characterization of *T. gondii* in Yunnan black goats in China. Thus, the objective of this present study was to determine the genotypes of *T. gondii* isolated from black goats in Yunnan province, southwest China, and the results would provide fundamental data for prevention and control of *T. gondii* infection in black goats.

## Methods

### Ethics statement

The collection of tissue samples from Yunnan black goats in this study was agreed by the abattoir manager. All animals were handled in strict accordance with good animal practice according to the Animal Ethics Procedures and Guidelines of the People’s Republic of China.

### Sample collection

In total, liver, lung and lymph nodes from 403 Yunnan black goats were collected randomly from different administrative regions in Yunnan province between June 2011 and March 2014, including 103 from Yuxi, 68 from Honghe, 85 from Kunming, 50 from Chuxiong and 97 from Qujing. Then, tissue samples were stored at −20°C prior to use.

### Genomic DNA extraction

Genomic DNA was extracted from different tissues using TIANamp Genomic DNA kit (TianGen™, Beijing, China) according to the manufacturer’s instructions. In brief, 50 mg of each tissue was treated with sodium dodecyl sulphate (200 μL) and proteinase K (20 μL) at 56°C for overnight digestion in a thermostat water bath. DNA samples were purified by silica gel column chromatography and obtained with 50 μL elution buffer.

### Genetic characterization of *T. gondii* isolates

The DNA samples of Yunnan black goats tissues were first examined for *T. gondii* infection by PCR amplification of B1gene [14] and then the positive samples were genotyped using Multi-locus PCR-RFLP (Mn-PCR-RFLP) method [15]. In brief, the target DNA sequences were amplified by multiplex PCR using external primers for all 10 markers. Then 1 μL of the products served as template DNA for nested PCR amplification with internal primers for each marker. The nested PCR products were digested with restriction enzymes for 3 h, at the corresponding temperature for each enzyme following the instruction for each enzyme. The restriction fragments were resolved in 2.5% agarose gel to display DNA fragment length polymorphism using a gel document system (UVP Gel Doc-It™ Imaging System, Cambridge, U.K.).

### Statistical analyses

The prevalence data were analyzed by Chi-squared tests using the program SPSS as previously (Release 19.0 standard version, SPSS Inc., Chicago, Illinois), and the probability (*P*) value <0.05 was considered statistically significant.

## Results and discussion

Out of 403 black goats, twenty (4.96%) were *T. gondii* B1 gene positive, and were distributed in all five administrative regions with the prevalence varying from 1.18% (Kunming) to 10.31% (Qujing), but the difference was not statistically significant (*P* > 0.05). The prevalence in different tissues ranged from 0.99% (lung) to 4.40% (lymph nodes), and the difference was statistically significant (*P* < 0.05) (Table 1). Previously, the overall prevalence of 65.79% [16] in hilar lymph nodes from pigs in south China, 7.83% [17] in hilar lymph nodes from pigs in central China, 10.98% [12] in liver from cats in southwest China, 53.85% [18] in lung from *Microtus fortis* in northeastern China were reported. Such significant differences in prevalence in various animals may due to several reasons, such as geographical origin, the tested number and the susceptibility to *T. gondii* of different animals.

**Table 1:** **Prevalence of** ***Toxoplasma gondii*** **infection in different tissues of Yunnan black goats detected by PCR**

**Category**	**Regions**					**Tissues**		
	**Yuxi**	**Honghe**	**Kunming**	**Chuxiong**	**Qujing**	**Liver**	**Lung**	**Lymph nodes**
Total no.	103	68	85	50	97	403	403	250
Positive no.	5	3	1	1	10	5	4	11
Prevalence (%)	4.85	4.41	1.18	2	10.31	1.24	0.99	4.40
Total prevalence (%)	4.96					1.89		

Due to the low DNA concentration, only 2 *T. gondii* isolates from Yunnan black goats presented complete genotyping data, and 6 *T. gondii* isolates were genotyped at 8 or more loci, whereas the rest 12 *T. gondii* isolates were genotyped by less than 6 loci, and considered unreliable, therefore not included for further analysis. Of these 8 *T. gondii* isolates with reliable typing data, two genotypes were revealed, namelyToxoDB#9 and ToxoDB#10 (Type I) (ToxoDB Version 11.0). Both genotypes were identified from Yunnan black goats in China for the first time, and these genotyping results were summarized in Table 2. Only one genotype (ToxoDB#9) was identified from Yuxi city in Yunnan province. This genotype has also been identified in previous studies in various animals: cats from Beijing Municipality, Guangdong, Anhui, Yunnan, Guizhou, Shandong, and Hubei provinces [12,19-23]; pigs from Guangdong, Henan, Yunnan and Anhui provinces [11,17,24,25]; and bats from Guangxi Zhuang Autonomous Region [13]. Therefore, ToxoDB#9 is a predominant lineage prevalent in Mainland China. Previous studies showed that ToxoDB#9 has been isolated from North and South America [26-29], as well as other Asian countries, such as Sri Lanka and Vietnam [30,31], indicating that it has a worldwide distribution.

**Table 2:** **Summary of genotyping of** ***Toxoplasma gondii*** **isolates from Yunnan black goats in different administrative areas of Yunnan province, southwest China**

**Isolate ID**	**Host**	**tissue**	**Location**	**SAG1**	**5’ + 3’ SAG2**	**Alternative SAG2**	**SAG3**	**BTUB**	**GRA6**	**c22-8**	**c29-2**	**L358**	**PK1**	**Apico**	**Genotype**
GT1	Goat		United States	I	I	I	I	I	I	I	I	I	I	I	Reference, Type I, ToxoDB#10
PTG	Sheep		United States	II/III	II	II	II	II	II	II	II	II	II	II	Reference, Type II, ToxoDB#1
CTG	Cat		United States	II/III	III	III	III	III	III	III	III	III	III	III	Reference, Type III, ToxoDB#2
MAS	Human		France	u-1	I	II	III	III	III	u-1	I	I	III	I	Reference, ToxoDB#17
TgCgCa1	Cougar		Canada	I	I	II	III	III	II	II	u-1	I	u-2	I	Reference, ToxoDB#66
TgCatBr5	Cat		Brazil	I	III	III	III	II	III	I	I	I	u-1	I	Reference, ToxoDB#19
TgWtdSc40	WTD		United States	u-1	II	II	II	II	II	II	II	I	II	I	Reference, Type 12, ToxoDB#5
TgCatBr64	Cat		Brazil	I	I	u-1	III	III	III	u-1	I	III	III	I	Reference, ToxoDB#111
TgRsCr1	Toucan		Costa Rica	u-1	I	II	III	I	III	u-2	I	I	III	I	Reference, ToxoDB#52
TgGYn1	BG	LN	Chuxiong, Yn	I	I	I	I	I	I	I	I	I	I	I	Type I, ToxoDB#10
TgGYn2	BG	Lung	Qujing, Yn	I	I	I	I	I	I	I	I	I	I	I	Type I, ToxoDB#10
TgGYn3	BG	LN	Qujing, Yn	I	I	I	I	I	I	I	I	I	nd	I	Type I, ToxoDB#10
TgGYn4	BG	Lung	Qujing, Yn	I	I	I	I	I	I	I	I	I	nd	I	Type I, ToxoDB#10
TgGYn5	BG	Liver	Qujing, Yn	I	I	I	I	I	I	I	I	I	nd	I	Type I, ToxoDB#10
TgGYn6	BG	Liver	Qujing, Yn	I	I	I	I	I	I	I	I	I	nd	I	Type I, ToxoDB#10
TgGYn7	BG	Liver	Kunming, Yn	I	I	I	I	nd	I	I	nd	I	I	I	Type I, ToxoDB#10
TgGYn8	BG	LN	Yuxi, Yn	u-1	II	II	III	III	II	II	nd	II	II	I	ToxoDB#9

u-1 and u-2 represent unique RFLP genotypes, respectively.

nd: no data. WTD: White-tailed Deer. BG: black goat. LN: Lymph nodes. Yn: Yunnan province.

In the present study, another genotype ToxoDB#10 (Type I) was identified from Yunnan black goats in Qujing, Chuxiong and Kunming of Yunnan province. Previously, this type was also found from humans in Shanghai city, Yunnan and Guangdong province [10,23,24], from cats in Yunnan province [12], from pigs in Hunan, Jiangsu and Henan provinces [17,24], and from bats in Yunnan and Guangxi [13], indicating that ToxoDB#10 is also a major lineage prevalent in Mainland China.

In this study, limited genetic diversity of *T. gondii* was found in black goats in Yunnan province. A relatively high frequency of the Type I strains found in black goats in this region is of interest. Since the Type I strain is highly virulent to mice and possibly more virulent to humans, future study to isolate viable parasite from this animal is necessary to confirm the finding.

## Conclusions

In summary, the present study revealed an overall prevalence of 4.96% *T. gondii* infection in Yunnan black goats by semi-nested PCR of B1 gene, and reported two *T. gondii* genotypes (ToxoDB#9 and Type I). To our knowledge, this is the first report of these genotypes in Yunnan black goats in China, which may have important implications for public health.
